# Chapter 15: Disease Gene Prioritization

**DOI:** 10.1371/journal.pcbi.1002902

**Published:** 2013-04-25

**Authors:** Yana Bromberg

**Affiliations:** Department of Biochemistry and Microbiology, School of Environmental and Biological Sciences, Rutgers University, New Brunswick, New Jersey, United States of America; (Whitehead Institute, United States of America), and Maricel Kann (University of Maryland, Baltimore County, United States of America)

## Abstract

Disease-causing aberrations in the normal function of a gene define that gene as a disease gene. Proving a causal link between a gene and a disease experimentally is expensive and time-consuming. Comprehensive prioritization of candidate genes prior to experimental testing drastically reduces the associated costs. Computational gene prioritization is based on various pieces of correlative evidence that associate each gene with the given disease and suggest possible causal links. A fair amount of this evidence comes from high-throughput experimentation. Thus, well-developed methods are necessary to reliably deal with the quantity of information at hand. Existing gene prioritization techniques already significantly improve the outcomes of targeted experimental studies. Faster and more reliable techniques that account for novel data types are necessary for the development of new diagnostics, treatments, and cure for many diseases.

This article is part of the “Translational Bioinformatics" collection for *PLOS Computational Biology*.

**What to Learn in This Chapter**

- Identification of specific disease genes is complicated by gene pleiotropy, polygenic nature of many diseases, varied influence of environmental factors, and overlying genome variation.
- Gene prioritization is the process of assigning likelihood of gene involvement in generating a disease phenotype. This approach narrows down, and arranges in the order of likelihood in disease involvement, the set of genes to be tested experimentally.
- The gene “priority" in disease is assigned by considering a set of relevant features such as gene expression and function, pathway involvement, and mutation effects.
- In general, disease genes tend to 1) interact with other disease genes, 2) harbor functionally deleterious mutations, 3) code for proteins localizing to the affected biological compartment (pathway, cellular space, or tissue), 4) have distinct sequence properties such as longer length and a higher number of exons, 5) have more orthologues and fewer paralogues.
- Data sources (directly experimental, extracted from knowledge-bases, or text-mining based) and mathematical/computational models used for gene prioritization vary widely.

## 1. Introduction

In 1904 Dr. James Herrick reported [1] the findings of “peculiar elongated and sickle shaped" red blood cells discovered by Dr. Ernest Irons in a hospital patient afflicted with shortness of breath, heart palpitations, and various other aches and pains. This was the first documented case of sickle cell disease in the United States. Forty years later, in 1949, sickle cell anemia became the first disease to be characterized on a molecular level [2], [3]. Thus, implicitly, the first disease-associated gene, coding for beta-globin chain of hemoglobin A, was discovered.

It took another thirty years before in 1983 a study of the DNA of families afflicted with Huntington's disease has revealed its association with a gene on chromosome 4 called huntigtin (HTT) [4]. Huntington's became the first genetic disease mapped using polymorphism information (G8 DNA probe/genetic marker), closely followed by the same year discovery of phenylketonuria association with polymorphisms in a hepatic enzyme phenylalanine hydroxylase [5]. These advances provided a route for predicting the likelihood of disease development and even stirred some worries regarding the possibility of the rise of “medical eugenics" [6]. Interestingly, it took another ten years for HTT's sequence to be identified and for the precise nature of the Huntigton's-associated mutation to be determined [7].

The recent explosion in high-throughput experimental techniques has contributed significantly to the identification of disease-associated genes and mutations. For instance, the latest release of SwissVar [8], a variation centered view of the Swiss-Prot database of genes and proteins [9], [10], reports nearly 20 thousand mutations in 35 hundred genes associated with over three thousand broad disease classes. Unfortunately, the improved efficiency in production of association data (*e.g.* genome-wide association studies, GWAS) has not been matched by its similarly improving accuracy. Thus, the sheer quantity of existing but yet unvalidated data resulted in information overflow. While association and linkage studies provide a lot of information, incorporation of other sources of evidence is necessary to narrow down the candidate search space. Computational methods - gene prioritization techniques, are therefore necessary to effectively translate the experimental data into legible disease-gene associations [11].

## 2. Background

The Merriam-Webster dictionary defines the word “disease" as a “a condition of the living animal or plant body or of one of its parts that impairs normal functioning and is typically manifested by distinguishing signs and symptoms." Thus, disease is defined *with respect to normal function* of said body or body part. Note, that this definition also describes the malfunction of individual cells or cell groups. In fact, many diseases can and should be defined on a cellular level. Understanding a disease, and potentially finding curative or preventive measures, requires answering three questions: (1) What is the affected function? (2) What functional activity levels are considered normal given the environmental contexts? (3) What is the direction and amount of change in this activity necessary to cause the observed phenotype?

Contrary to the view that historically prevailed in classical genetics it is rarely the case that one gene is responsible for one function. Rather, an assembly of genes constitutes a functional module or a molecular pathway. By definition, a molecular pathway leads to some specific end point in cellular functionality via a series of interactions between molecules in the cell. Alterations in any of the normally occurring processes, molecular interactions, and pathways lead to disease. For example, folate metabolism is an important molecular pathway, the disruptions in which have been associated with many disorders including colorectal cancer [12] and coronary heart disease [13]. Because this pathway involves 19 proteins interacting via numerous cycles and feedback loops [14], it is not surprising that there are a number of different ways in which it can be broken. The changes in concentrations and/or activity levels of any of the pathway members directly affect the pathway end-products (*e.g.* pyrimidine and/or methylated DNA). The specifics of a given change define the severity and the type of the resulting disease; see Box 1 for discussion on disease types. Moreover, since the view of a single pathway as a discrete and independent entity (with no overlap with other pathways) is an oversimplification, it is increasingly evident that different diseases are also interdependent.

**Box 1. Genetic similarities of different disease types**

Diseases can be very generally classified by their associated causes: *pathogenic* (caused by an infection), *environmentally determined* (caused by “inanimate" environmental stressors and deficiencies, such as physical trauma, nutrient deficiency, radiation exposure and sleep deprivation), and *genetically hereditary* or *spontaneous* (defined by germline mutations and spontaneous errors in DNA transcription, respectively). Moreover, certain genotypes are more susceptible to the effects of pathogens and environmental stress, contributing to a deadly interplay between disease causes. Regardless of the cause of disease, its manifestations are defined by the changes in the affected function. For example, cancer is the result of DNA damage occurring in a normal cell and leading toward a growth and survival advantage. The initial damage is generally limited to a fairly small number of mutations in key genes, such as proto-oncogenes and tumor suppressor genes [135]. The method of accumulation of these mutants is not very important. A viral infection may cause cancer by enhancing proto-oncogene function [136] or by inserting viral oncogenes into host cell genome. An inherited genetic variant may disrupt or silence a single allele of a mismatch-repair gene as in Lynch syndrome [137]. Spontaneous transcription errors and influence of environmental factors, *e.g.* continued exposure to high levels of ionizing radiation, may result in oncogene and tumor suppressor-gene mutations leading to the development of cancer [138]. Thus, the same broad types of disease can be caused by the disruption of the same mechanisms or pathways resulting from any of the three types of causes.

## 3. Interpreting What We Know

Identifying the genetic underpinnings of the observed disease is a major challenge in human genetics. Since disease results from the alteration of normal function, identifying disease genes requires defining molecular pathways whose disrupted functionality is necessary and sufficient to cause the observed disease. The pathway function changes due to the (1) changes in gene expression (*i.e.* quantity and concentration of product), (2) changes in structure of the gene-product (*e.g.* conformational change, binding site obstruction, loss of ligand affinity, etc.), (3) introduction of new pathway members (*e.g.* activation of previously silent genes), and (4) environmental disruptions (*e.g.* increased temperatures due to inflammation or decreased ligand concentrations due to malnutrition). While all members of the affected pathways can be construed as disease genes, the identification of a subset of the true causative culprits is difficult. Obscuring such identification are individual genome variation (*i.e.* the reference definition of “normal" is person-specific), multigenic nature and complex phenotypes of most diseases, varied influence of environmental factors, as well as experimental data heterogeneity and constraints.

**Figure 1:**
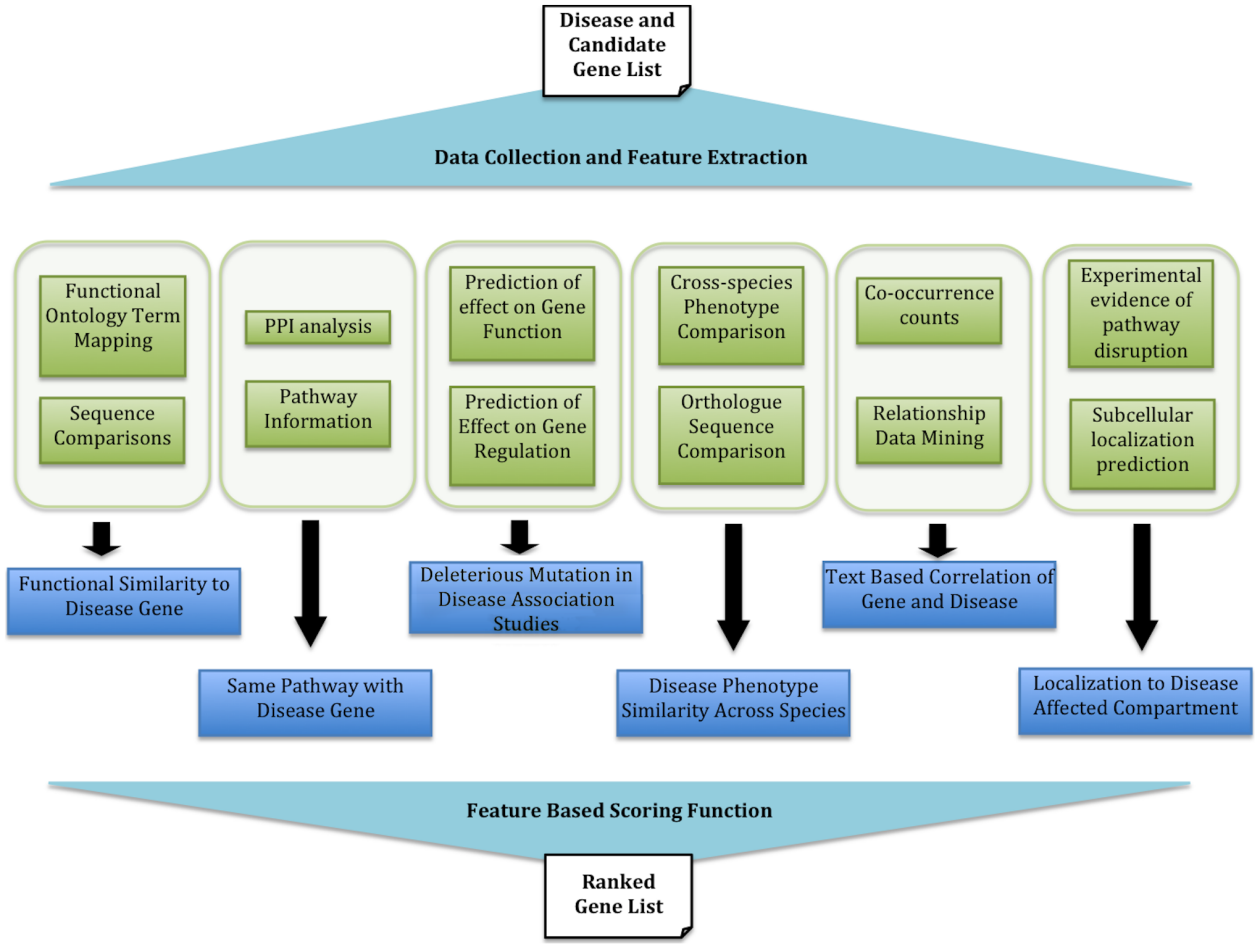
Overview of gene prioritization data flow. In order to prioritize disease-gene candidates various pieces of information about the disease and the candidate genetic interval are collected (green layer). These describe the biological relationships and concepts (blue layer) relating the disease to the possible causal genes. Note, the blue layer (representing the biological meaning) should ideally be blind to the content green layer (information collection); *i.e.* any resource that describes the needed concepts may be used by a gene prioritization method.

Disease genes are most often identified using: (1) genome wide association or linkage analysis studies, (2) similarity or linkage to and co-regulation/co-expression/co-localization with known disease genes, and (3) participation in known disease-associated pathways or compartments. In bioinformatics, these are represented by multiple sources of evidence, both direct, *i.e.* evidence coming from own experimental work and from literature, and indirect, *i.e.* “guilt-by-association" data. The latter means that genes that are in any way related to already established disease-associated genes are promoted in the suspect list. Additionally, implied gene-disease links, such as functional deleteriousness of mutations affecting candidate genes, contributes to establishing associations. The manner in which each guilty association is derived varies from tool to tool and all of them deserve consideration. Very broadly, gene-disease associations are inferred from (Figure 1):

1. *Functional Evidence* – the suspect gene is a member of the same molecular pathways as other disease-genes; inferred from: direct molecular interactions, transcriptional co-(regulation/expression/localization), genetic linkage, sequence/structure similarity, and paralogy (in-species homology resulting from a gene duplication event)
2. *Cross-species Evidence* – the suspect gene has homologues implicated in generating similar phenotypes in other organisms
3. *Same-compartment Evidence* – the suspect gene is active in disease-associated pathways (*e.g.* ion channels), cellular compartments (*e.g.* cell membrane), and tissues (*e.g.* liver).
4. *Mutation Evidence* – suspect genes are affected by functionally deleterious mutations in genomes of diseased individuals
5. *Text Evidence* – there is ample co-occurrence of gene and disease terms in scientific texts. Note that textual co-occurrence represents some form of biological evidence, which does not yet lend itself to explicit documentation.

### 3.1 Functional Evidence

#### 3.1.1 Molecular interactions

**Figure 2:**
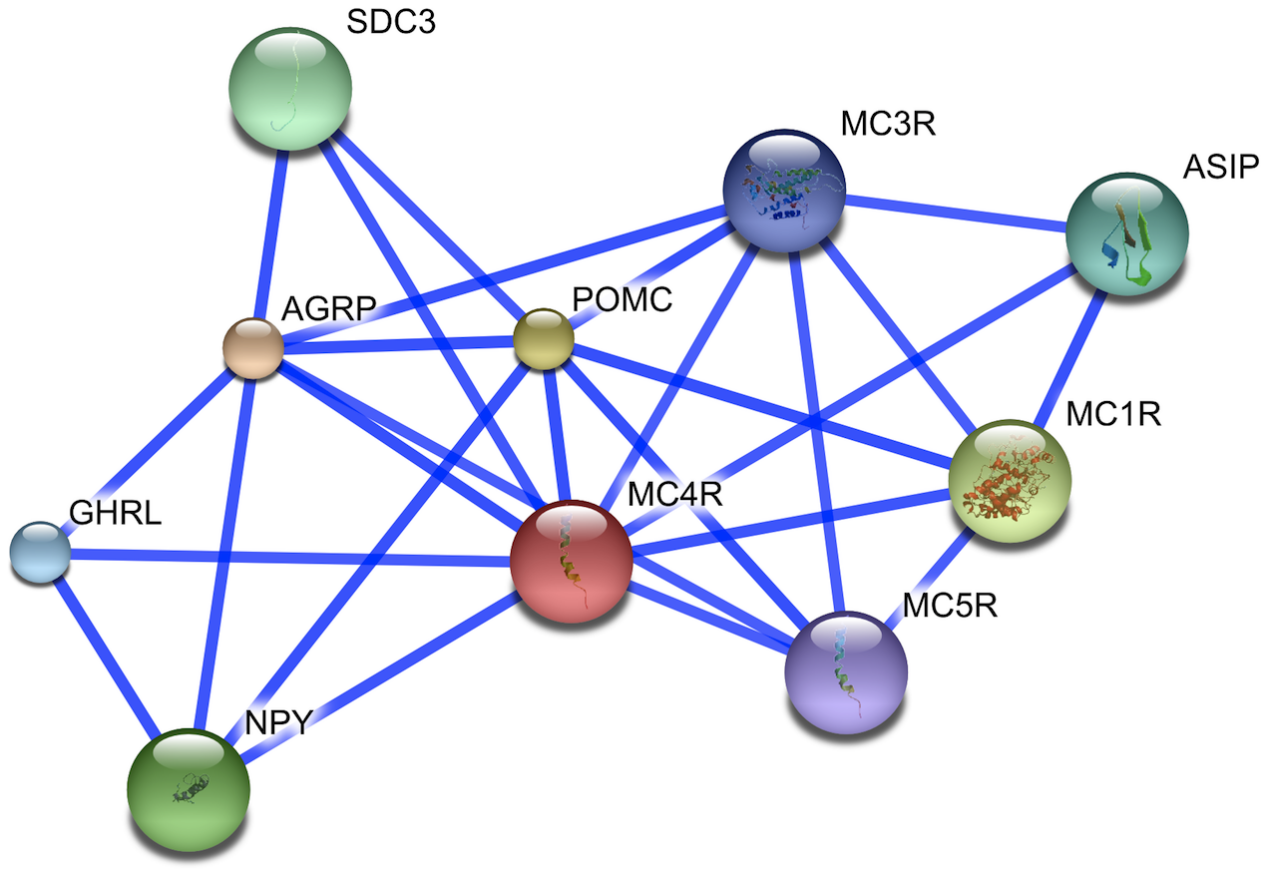
MC4R-centered protein-protein interaction network. The figure illustrates protein-protein interaction neighborhood of the human melanocortin 4 receptor (MC4R) as illustrated by the confidence view of the STRING 8.3 server. The nodes of the graph represent human proteins and the connections illustrate their known or predicted, direct and indirect interactions. The connection between any two protein-nodes is based on the available information mined from relevant databases and literature. The network includes all protein interactions that have >0.9 estimated probability.

Gene prioritization tools, from the earliest field pioneers like G2D [15], [16], [17] to the more recent ENDEAVOUR [18], [19] and GeneWanderer [20], [21], among many others, have used gene-gene (protein-protein) interaction and/or pathway information to prioritize candidate genes. Biologically this makes sense, because if diseases result from pathway breakdown then disabling any of the pathway components can produce similar phenotypes; *i.e.* genes responsible for similar diseases often participate in the same interaction networks [22], [23]. To illustrate this point, consider the interaction partners of the melanocortin 4 receptor (MC4R) in STRING [24], [25] server generated Figure 2. Note, not all known interactions are shown – the inclusion parameter is STRING server likelihood >0.9.

MC4R is a hypothalamic receptor with a primary function of energy homeostasis and food intake regulation. Functionally deleterious polymorphisms in this receptor are known to be associated with severe obesity [26], [27], [28]. Here, MC1R, MC3R, and MC5R are membrane bound melanocortin (1,3,5) receptors that interact with MC4R via shared binding partners. Syndecan-3 (SDC3), agouti signaling protein precursor (ASIP), agouti related protein precursor (AgRP), pro-opiomelanocortin (POMC) and/or their processed derivatives directly bind MC4R for varied purposes of the MC4R signaling pathway. Finally, the reported interactions with Neuropeptide Y-precursor (NPY) and the growth hormone releasing protein (GHRL) are literature derived and may reflect indirect, but tight connectivity. By the token of “same pathway" evidence, *MC4R* interactors, whether agonists or antagonists, may be predicted to be linked to obesity. In fact, mutations that negatively affect normal POMC production or processing have been shown to be obesity-associated [29], [30] and gene association studies have linked AgRP with anorexia and bulimia nervosa behavioral traits [31], representative of food intake abnormalities. Other pathway participants have also been marked and extensively studied for obesity association.

#### 3.1.2 Regulatory and genetic linkage

Co-regulation of genes has traditionally been thought to point to their involvement in same molecular pathways [32] and, by that token, to similar disease phenotypes; *e.g.* [33], [34]. For example, GPR30 a novel G-protein coupled estrogen receptor is co-expressed with the classical estrogen receptor ERβ [33]. The former (GPR30) has been linked to endometrial carcinoma [35] so it is no surprise that the latter (ERβ) is also associated with this type of cancer [33].

However, co-regulation doesn't *always* have to mean the same pathway – studies have shown that consistently co-expressed genes, while possibly genetically linked [36], [37], may also reside in distinct pathways [38]. Additionally, co-expressed non-paralogous genes, independent of common pathway involvement, often cluster together in different species and fall into chromosomal regions with low recombination rates [39], [40], suggesting genetic linkage [39], [40]. These finding suggests that clusters of co-expressed genes are selectively advantageous [36]. Possibly, these clusters are groups of genes that despite the apparent functional heterogeneity may be jointly involved in orchestrating complicated cellular functionality [41]. Evolutionary pressure works on maintaining co-expression of these genes and on keeping recombination rates within the clusters low. Thus, the fine-tuned cooperation of alleles is not broken by recombination, but rather transmitted as one entity to the next generation. De-regulation of these clusters is therefore likely to be deleterious to the organism and develop into disease.

Genes co-expressed with or genetically linked to other disease genes are also likely to be disease-associated. However, while genetic linkage and co-regulation are valuable markers of disease association, they also pose a specificity problem; *i.e.* a given disease-associated gene may be co-regulated with or linked to another disease-associated gene, where the two diseases are not identical. Genetic linkage similarly poses a problem for GWAS where it is difficult to distinguish between “driver" mutations, the actual causes of disease, and “passenger" mutations, co-occurring with the disease-mutations due to genetic linkage.

#### 3.1.3 Similar sequence/structure/function

Reduced or absent phenotypic effect in response to gene knockout/inactivation is a common occurrence [42], [43], largely explained by functional compensation, *i.e.* partial interchangeability of paralogous genes. In humans, genes with at least one paralogue, approximated by 90% sequence identity, are about three times less likely to be associated with disease as compared to genes with more remote homologs [44]. However, in the cases where paralogous functional compensation is insufficient to restore normal function, inactivation of any of the paralogues leads to same or similar disease. Prioritization tools thus often use functional similarity as an input feature. For example, one GeneOntology (GO, [45]) defined MC4R function, is “melanocyte-stimulating hormone receptor activity" (GO:0004980). There are two other human gene products sharing this function: MSHR (MC1R, 52% sequence identity) and MC3R (61%). Predictors relying on functional similarity to annotate disease association would inevitably link both of these with obesity. These findings are confirmed by the recent studies for MC3R [46], but the jury still remains out for MC1R involvement.

Quantifying functional similarity is of utmost importance for the above approach. Using ontology-defined functions (*e.g.* GeneOntology) this problem reduces to finding a distance between two ontology nodes/subtrees (*e.g.* [47], [48], [49], [50]). For un-annotated genes, however, sequence and structure homology is often used to transfer functional annotations from studied genes and proteins [51], [52]. Since functionally similar genes are likely to produce similar disease phenotypes, sequence/structure similarities are good indicators of similar disease involvement. Additionally, disease genes are often associated with specific gene and protein features such as higher exon number and longer gene length, protein length, presence of signal peptides, higher distance to a neighboring gene and 3′ UTR length, and lower sequence divergence from their orthologues [53], [54]. Moreover, disordered proteins are often implicated in cancer [55].

### 3.2 Cross-species Evidence

Animal models exist for a broad range of human diseases in a number of well-studied laboratory organisms, *i.e.* mouse, zebrafish, fruit fly, etc. However, straightforward cross-species comparisons of orthologues and their associated phenotypic traits are also very useful. A high number of orthologues (consistent presence in multiple species) generally highlights essential genes that are prone to disease involvement. Orthologues generally participate in similar molecular pathways although different levels of function are necessary for different organisms (*e.g.* human MC4R is more functional then its polar bear orthologue [56]). Thus, cross-species tissue-specific phenotypic differentiation due to slightly varied sequences may be useful for gene prioritization. For example, the human MC4R and almost all of its close orthologues (*e.g.* in mouse, rat, pig, and cow) contain a conserved valine residue in the 95^th^ position of the amino acid sequence. In the polar bear orthologue, however, this position is frequently occupied by an isoleucine residue [56]. When considering MC4R involvement in generating an obesity phenotype, it is useful to note that polar bears have a need for increased body fat content for thermal insulation, water buoyancy, and energy storage requirements [56] as compared to humans and to other organisms that share a conserved V95. Thus, one can imagine that the V95I mutation, while deleterious to the function of the receptor, is a polar bear specific adaptation to its environment, and may have a similar (increased body fat) effect in humans. In fact, V95I does inactivate the human receptor [57], [58] and associates with obesity.

**Figure 3:**
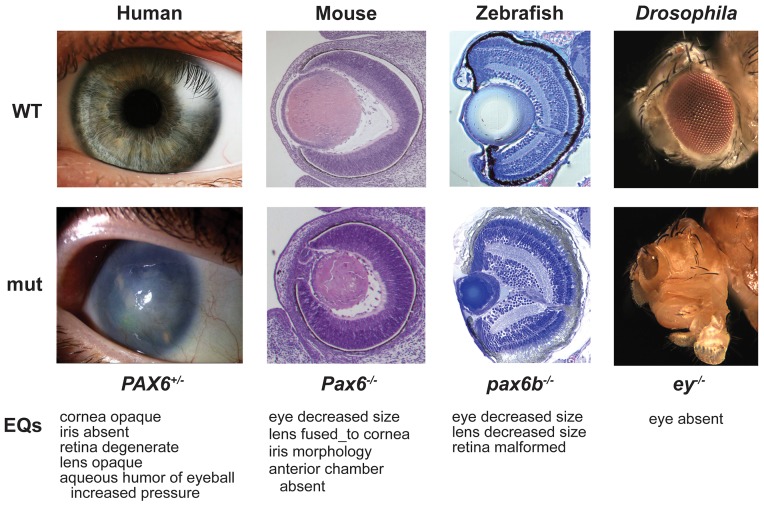
Correlating cross-species phenotypes. Phenotypes of wild-type (top) and PAX6 ortholog mutations (bottom) in human, mouse, zebrafish, and fly can be described with the EQ method suggested by Washington et al [59]. Once phenotypic descriptions are standardized across species, genotypic variations can be assessed as well.

Comparing human and animal phenotypes is not always straightforward. Washington *et al* [59] have shown that phenotype ontologies facilitate genotype-phenotype comparisons across species. Disease phenotypes recorded in their ontology (OBD, ontology based database) can be compared to the similarly built cross-species phenotype ontologies using a set of proposed similarity metrics. Finding related phenotypes across species suggests orthologous human candidate genes. For instance, phenotypic similarities of eye abnormalities recorded in human and fly suggest that *PAX6*, a human orthologue of the phenotype-associated fly gene *ey*, is a possible disease-gene candidate. Further investigation shows that mutations in *PAX6* may result in aniridia (absence of iris), corneal opacity (aniridia-related keratopathy), cataract (lens clouding), glaucoma, and long-term retinal degeneration (Figure 3) [59].

A correlation of gene co-expression across species is also useful for annotating disease genes [60], [61]. Genes that are part of the same functional module are generally co-expressed. Also, there is evidence for co-expression of visibly functionally unrelated genes [37], [62], [63]. The explanation of these co-expression clusters having an evolutionary advantage only holds true for otherwise unjustified conservation of these clusters throughout different species; *i.e.* cross-species comparison of protein co-expression may be used for validation of disease-gene co-expression inference. Using this assumption, Ala *et al* [61] had narrowed down the initial list of 1,762 genes in the loci mapped via genetic linkage to 850 OMIM (Online Mendelian Inheritance in Man) [64] phenotypes to twenty times fewer (81) possible disease-causing genes. For example, in their analysis a cluster of functionally unrelated genes co-expressed in human and mouse contained a *bona fide* disease-gene KCNIP4 (partial epilepsy with pericentral spikes).

### 3.3 Compartment Evidence

Changes in gene expression in disease-affected tissues are associated with many complex diseases [65]. Tissue specificity is also important for choosing correct protein-protein interaction networks, as some proteins interact in some tissues, but rarely in others [66]. Disease-associated cellular pathways (*e.g.* ion channels or endocytic membrane transport) and compartments (*e.g.* membrane or nucleus) implicate pathway/compartment-specific gene-products in disease as well. For example, autosomal recessive generalized myotonia (Becker's disease) (GM) and autosomal dominant myotoniacongenita (Thomsen's disease, MC) are characterized by skeletal muscle stiffness [67]. This phenotype is the result of muscle membrane hyperexcitability and, in conjunction with observed alterations in muscle chloride and sodium currents, points to possible involvement of deficiencies of the muscle chloride channel. In fact, studies point to the mutations in the transmembrane region of CLC-1, the muscle chloride channel coding gene, as the culprit [67]. Another example is that of the multiple storage diseases, such as Tay-Sachs, Gaucher, Niemann-Pick and Pompe disease, which are caused by the impairment of the degradation pathways of the intracellular vesicular transport. In fact, many of the genes implicated in these diseases encode for proteins localized to endosomes (*e.g. NPC1* in Neimann-Pick [68]) or lysosomes (*e.g. GBA* [69] in Gaucher, *GAA* in Pompe [70] and *HEXA* in Tay Sachs [71]).

### 3.4 Mutant Evidence

By definition, every genetic disease is associated with some sort of mutation that alters normal functionality. In fact, primary selection of candidates for further analysis is often largely based on observations of polymorphisms in diseased individuals, which are absent in healthy controls (*e.g.* GWAS). However, not all observed polymorphisms are associated with deleterious effects. Note, that on average gain and loss of function mutations are considered to alter normal functionality equally deleteriously. Most of the observed variation does not at all manifest phenotypically, some is weakly deleterious with respect to normal function, and less still is weakly beneficial. In nature strongly beneficial mutations are very rare; they spread rapidly in the population and cannot be considered disease-associated. On the other hand, strongly deleterious or inactivating mutations are often incompatible with life. A small percentage of mutations of this type, affecting genes whose function is not life-essential, are often associated with monogenic Mendelian disorders. Strongly deleterious mutations in the genes whose function may somehow be compensated (*e.g.* via paralogue activity) are associated with complex disorders, where the level of compensation affects the observed phenotype. Complex disorders may also accumulate weakly deleterious mutations to generate a strongly negative phenotype. Intuitively it is clear that a selected candidate gene, carrying a deleterious mutation in an affected individual is more likely to be disease-associated than one which contains functionally neutral mutants or no variation at all.

#### 3.4.1 Structural variation

Structural variation (SV) is the least studied of all types of mutations. It has long been assumed that less than 10% of human genetic variation is in the form of genome structural variants (insertions and deletions, inversions, translocations, aneuploidy, and copy number variations - CNVs). However, because each of the structural variants is large (kb-Mb scale), the total number of base pairs affected by SVs may actually be comparable to the number of base pairs affected by the much more common SNPs (single nucleotide polymorphisms). Moreover, high throughput detection of structural variants is notoriously difficult and is only now becoming possible with better sequencing techniques and CNV arrays. Thus, more SVs may be discovered in the near future. We do not currently know what proportion of genetic disease is caused by SVs, but we suspect that it is high.

Due to the above mentioned constraints on SV identification, there are only ∼180 thousand structural variants reported in one of the most complete mutation collections – the Database of Genomic Variants, DGV [72]. Gross changes to genome sequence are very likely to be disease associated, but also frequently gene non-specific. For instance, Down's syndrome, trisomy 21, is an example of a whole extra chromosome gain and *cri du chat* syndrome results from the deletion of the short arm of chromosome 5 [73]. All of the genes found in these regions of the genome are, by default, associated with the observed disease but neither can be considered primarily causal. When the damage is less extensive the genes involved may be further evaluated for causation. For instance, several epilepsy-associated genes are known, but functionally-significant mutations in these account for only a small fraction of observed disease cases. One study [74] reports that CNV mutants found in epileptic individuals but not in the general population account for nearly nine percent of all cases. Among these are CNVs resulting from deletions in AUTS2 and CNTNAP2 genes. Both of these genes have been implicated in other neurological disorders [75], [76] reaffirming the possible disease link. Inversions, translocations and large deletions and insertions have all been implicated in different forms of disease. Even very small indels, resulting in an open-reading frame shift (frameshift mutations), are often sufficient to cause disease. For instance, one of the causes of Tay-Sachs is a deletion of a single cytosine nucleotide in the coding sequence of a lysosomal enzyme beta-hexosaminidase [71].

In most cases of diseases that are associated with SVs the prioritization of disease-causing genes is reduced to finding those that are directly affected by the mutation. Lots of work has been done in this direction, including development of the CNVinetta package [77] for mining and visualizing CNVs, GASV approach for identifying structural variation boundaries more precisely [78], and software created by Ritz *et al* for searching for structural variants in strobe sequencing data [79]. SV identification is still a new field, but the advances in methodologies will have a great impact on our understanding and study of many of the known diseases.

#### 3.4.2 Nucleotide polymorphisms

The other ∼90% of human variation exists in the form of SNPs (single nucleotide polymorphisms) and MNPs (multi-nucleotide polymorphisms; consecutive nucleotide substitutions, usually of length two or three). A single human genome is expected to contain roughly 10–15 million SNPs per person [80]. As many as 93% of all human genes contain at least one SNP and 98% of all genes are in the vicinity (∼5 kb) of a SNP [81]. The latest release of NCBI dbSNP database [82] (build 137) contains nearly 43 million validated human SNPs, 17.5 million of which have been experimentally mapped to functionally distinct regions of the genome (*i.e.* mRNA UTR, intron, or coding regions). Non-coding region SNPs (∼17.2 million) are trivially more prevalent than coding SNPs (∼432 thousand) as non-coding DNA makes up the vast majority of the genome. Coding SNPs, however, are over-represented in disease associations; *e.g.* OMIM contains 2430 non-coding SNPs (0.0001% of all) and 5327 coding ones (0.01% of all – 100-fold enrichment). Due to the redundancy of the genetic code, coding SNPs can be further subdivided into synonymous (no effect on protein sequence) and non-synonymous (single amino acid substitution) SNPs. Simple statistics of the genetic code suggest that synonymous SNPs should account for 24% of all coding-region SNPs. dbSNP data suggests an even larger percentage of synonymity – ∼188 thousand (44%), which is possibly due to evolutionary pressure eliminating functionally deleterious non-synonymous SNPs. MNPs are rare as compared to SNPs, but are over-represented amongst the protein altering variants, almost always changing the affected amino acid, or two neighboring ones, or introducing a nonsense mutation (stop-codon) [83].

Identifying and annotating functional effects of SNPs and MNPs is important in the context of gene prioritization because genes selected for further disease-association studies are more likely to contain a deleterious mutation or be under the control of one (*e.g.* mutations affecting transcription factor or microRNA binding sites). In recent years a number of methods were created for identifying mutations as functionally deleterious. PromoLign [84], PupaSNP finder [85], and RAVEN [86] look for SNPs affecting transcription, SNPper [87] finds and annotates SNP locations, conservation, and possible functionalities so that they can be visually assessed, and SNPselector [88] and FASTSNP [89] assess various SNP features such as whether it alters the binding site of a transcription factor, affects the promoter/regulatory region, damages the 3′ UTR sequence that may affect post-transcriptional regulation, or eliminates a necessary splice site. Coding synonymous SNPs have recently been shown to have the same chance of being involved in a disease mechanism as non-coding SNPs [90]. This effect may be due to codon usage bias or to changes in splicing or miRNA binding sites [91]. However, few (if any) computational methods are able make predictions with regard to their functional effects.

Non-synonymous SNPs are somewhat more studied. Early termination of the protein is very often associated with disease so genes with nonsense mutants are automatically moved up in the list of possible suspects. Missense SNPs and MNPs, which alter the protein sequence without destroying it, may or may not be disease associated. In fact, most methods estimate that only 25–30% of the nsSNPs negatively affect protein function [92]. Databases like OMIM [93], and more explicitly, SNPdbe [94], SNPeffect [95], PolyDoms [96], Mutation@A Glance [97] and DMDM [98] map SNPs to known structural/functional effects and diseases. Computational tools that make predictions about functional and disease-associated effects of SNPs include SNAP [99], [100], SIFT [101], [102], PolyPhen [103], [104], PHD-SNP [105], SNPs3D [106], and many others. Most of these methods are binary in essence – that is they point to a deficiency without suggesting specifics of the disease or molecular mechanisms of functional failure. Nevertheless, they are very useful in conjunction with other data described above. The recent trend in mutation analysis has seen the development of tools, like SNPNexus [107] and SNPEffectPredictor [108] that are no longer limited by DNA type and predict effects for both non-coding and coding region SNPs.

### 3.5 Text Evidence

The body of science that addresses gene-disease associations has been growing in leaps and bounds since the mapping of a hemoglobin mutation to sickle cell anemia. Some researchers have been proactive in making their data computationally available from databases like dbSNP, GAD [109], COSMIC [110], *etc*. Others have contributed by depositing knowledge obtained through reading and manual curation into the likes of PMD [111], GeneRIF [112] and UniProt [9]. However, huge amounts of data, which could potentially improve the performance of any gene prioritization method, remains hidden in plain site in natural language text of scientific publications. Consider, for example, a scientist who is interested in prioritizing breast cancer genes. A casual search in PubMed for the term combination *breast cancer* generates over two hundred thousand matches. Limiting the field to *genetics of breast cancer* reduces the count to slightly fewer than fifty thousand. The past thirty days have brought about 46 new papers. Thus, someone interested in getting all the genetic information out of the PubMed collection would need to dedicate his or her life to reading. Fortunately, scientific text mining tools have recently come of age [113], [114], [115]. The new tools will allow for intelligent identification of possible gene-gene and disease-gene correlations [116], [117], [118]. For example, the Information Hyperlinked Over Proteins, IHOP method [119] links gene/protein names in scientific texts via associated phenotypes and interaction information. For automated link extraction, however, the existing gene prioritization techniques rely mostly on term co-occurrence statistics (*e.g.* PosMed [120] and GeneDistiller [121]) and gene-function annotations (*e.g.* ENDEAVOR [122] and PolySearch [123]), which can then be related to diseases as described above.

**Figure 4:**
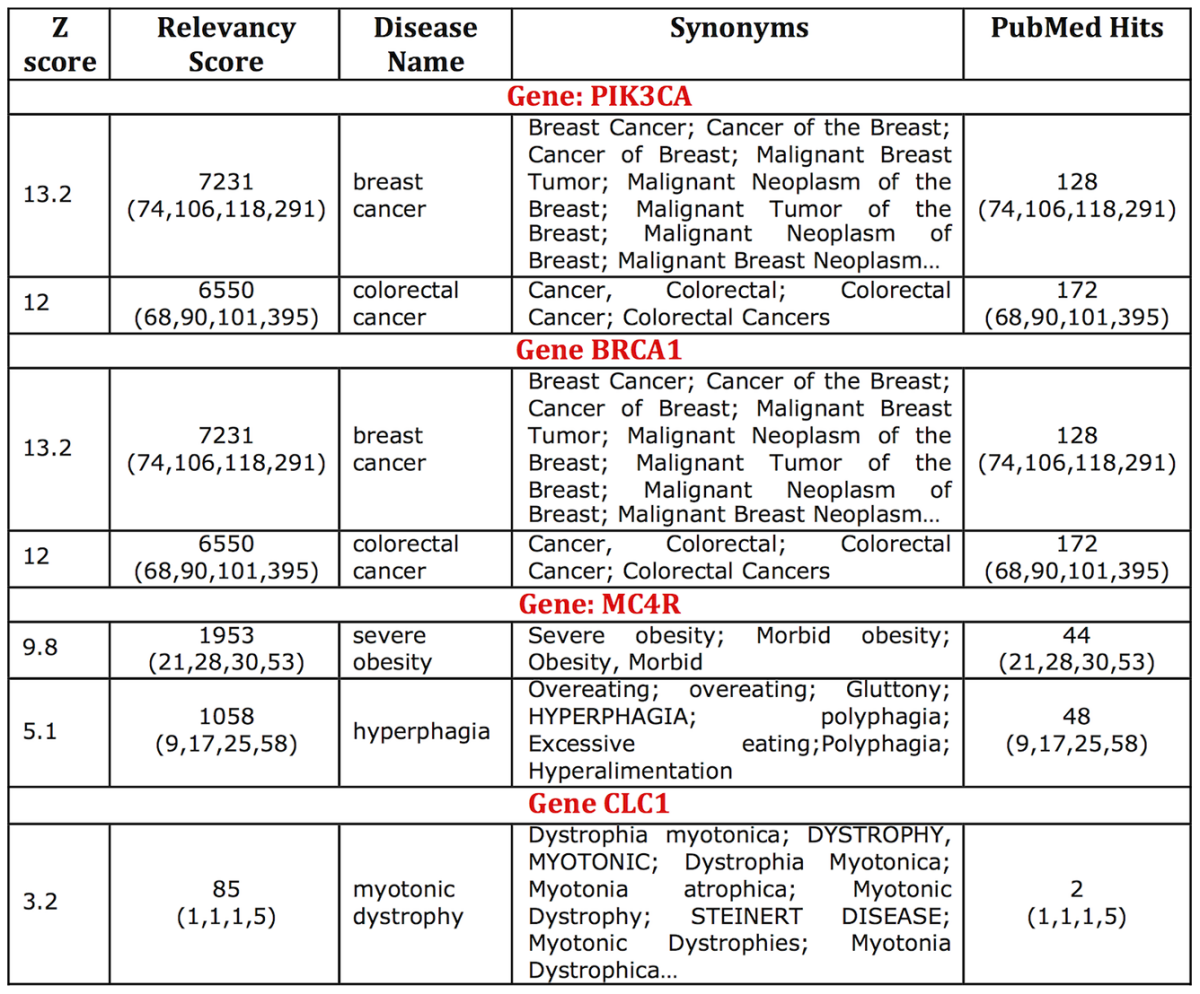
PolySearch gene-disease associations. PolySearch uses PubMed lookup results to prioritize diseases associated with a given gene. Here, screen shots of the top two results (where available; sorted by relevancy score metric) from PolySearch are shown. According to these, BRCA1 and PIK3CA are associated with breast cancer, while MC4R and CLC1 are not. These results quantitatively confirm intuitive inferences made from simple PubMed searches.

For a significantly oversimplified example of this type of processing consider searching PubMed for the terms *breast cancer* and *BRCA1*. The initial search returns 50 articles, as compared to 21 for *breast cancer* with *BRCA2*, 6 with *PIK3CA*, 1 with *TOX3*, and 0 for *MC4R* or *CLC1* associations. While the number of publications reflects many extraneous factors such as the popularity and “research age" of the protein, it is also very much reflective of the possibility of gene-disease association. Thus, BRCA1 and BRCA2 would be the most likely candidates for cancer association, followed by PIK3CA and TOX3. MC4R and CLC1 would not make the cut. Note that PubMed now defaults to a smart search engine, which identifies all aliases of the gene and the disease while cutting out more promiscuous matches; *i.e.* turning off the translation of terms would result in significantly more less accurate matches. Using specialized tools like PolySearch (or IHOP) to perform the same queries produces more refined and quantifiable results (Figure 4).

## 4. The Inputs and Outputs

Existing disease-gene prioritization methods vary based on the types of inputs that they use to produce their varied outputs. Functionality of prioritization methods is defined by previously known information about the disease and by candidate search space [124], which may be either submitted by the user or automatically selected by the tool. Disease information is generally limited to lists of known disease-associated genes, affected tissues and pathways and relevant keywords. The candidate search space does not have to be input at all (*i.e.* the entire genome) or be defined by the suspect (for varied experimental reasons) genomic region. The prioritization accuracy, in large part, depends on the accuracy and specificity of the inputs. Thus, providing a list of very broad keywords may reduce the performance specificity, while incorrect candidate search space automatically decreases sensitivity. Prioritization methods generally output ranked/ordered lists of genes, oftentimes associated with p-values, classifier scores, etc.

Overall, input and output requirements and formats are a very important part of establishing a tool's relevance for its users. As with other bioinformatics methods, the ease use and the steepness of learning curve for a given gene prioritization method often define the user base at least as strictly as does its performance.

## 5. The Processing

Gene prioritization methods use different algorithms to make sense of all the data they extract, including mathematical/statistical models/methods (*e.g.* GeneProspector [125]), fuzzy logic (*e.g.* ToppGene [126], [127]), and artificial learning devices (*e.g.* PROSPECTR [54]), among others. Some methods use combinations of the above. Objectively, there is no one methodology that is better than the others for all data inputs. For more details on computational methods used in the various approaches please refer to relevant tool publications and method-specific computer science/mathematics literature, *e.g.* [128], [129], [130], [131], [132], [133], [134].

**Figure 5:**
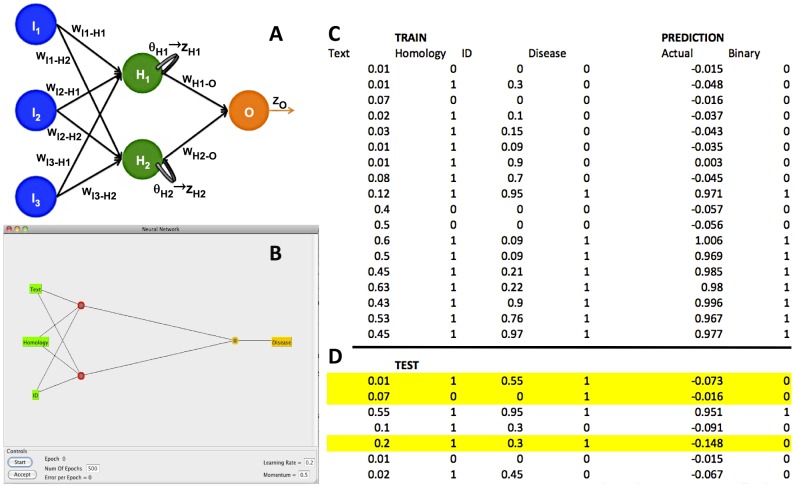
Predicting gene-disease involvement using artificial neural networks (ANNs). In a supervised learning paradigm, the neural networks are trained using experimental data correlating inputs (descriptive features relating genes to diseases) to outputs (likelihood of gene-disease involvement). The training and testing procedures for the generalized network (Panel A) are described in text. In our example, the WEKA [129], [130], [131], [139] ANN (Panel B; a = 0.5, λ = 0.2) is trained using the training set (Panel C) repeated 500 times (epochs). The network “memorizes" (Predictions in Panel C) the patterns in the training set and is capable of making accurate predictions for four out of seven instances it has not seen before (test set, Panel D). It is important to note here that the erroneously assigned instances (yellow highlight) in the test set are, for the most part, unlike the training. The first one has very little literature correlation (0.01), while sequence similarity to another disease-involved gene is fairly high 0.55). The second maps an unlikely candidate gene (very low literature, no homology) to disease, and the third has barely enough literature mapping and borderline homology. Representation of neither of these instances was consistently present in the training set. This example highlights the importance of training using a representative training set, while testing on a set that is not equivalent to training.

To illustrate the general concepts of relying on the various computational techniques for gene prioritization we will consider the use of an artificial neural network (ANN). Keep in mind that while methods and their requirements differ, the notion of identifying patterns in the data that may be indicative disease-gene involvement remains the same throughout. In simplest terms, a neural network is essentially a mathematical model that defines a function *f*: *X*→*Y*, where a distribution over X (the inputs to the network) is mapped to a distribution over *Y* (the outputs/classifications). The word “network" in the name “artificial neural network" refers to the set of connections between the “neurons" (Figure 5). The functionality of the network is defined by the transmission of signal from activated neurons in one layer to the neurons in another layer via established (and weighed) connections. Besides the choice and number of inputs and outputs, the parameters defining a given ANN are (1) interconnection patterns, (2) the process by which the weights of connections are selected/updated (learning function), and (3) the activation thresholds (functions) of any one given neuron. “Training" a network means optimizing these parameters using an existing set of inputs (and, possibly, outputs). Ultimately, a trained network could then relatively accurately recognize learned patterns in previously unseen data. For more details regarding the possible types and parameters of neural networks see [132], [134]. For an illustration of network application see Box 2 and Figure 5.

**Box 2. Illustrating basic functionality of a standard (on-line fully-interconnected feed-forward sigmoid-function back-propagating) neural network**

In Figure 5A example network there are three fully interconnected layers of neurons (input, hidden, and output layers); *i.e.* each neuron in one layer is connected to every neuron in the next layer. The three input neurons encode biologically relevant pieces of data relating a given gene G to a given disease D. For each G and D, *i_neuron1* is the fraction of articles (out of 1000) containing in-text co-occurrences of G and D and *i_neuron2* represents the presence/absence of a sequence-similar gene G' associated with D (*i_neuron3* = G/G' sequence identity). The hidden (inference) layer consists of two neurons *h_neuron1* and *h_neuron2* with activation thresholds θ_1_ and θ_2_, respectively. The single output, *o_neuron* (threshold θ_O_) represents the involvement of G in causing D: 0 = no involvement, 1 = direct causation. The starting weights of the network (w_i1-h1_, w_i1-h2_…w_h2-o_) are arbitrarily assigned random values between 0 and 1. Intuitively, the function of the network is to convert input neuron values into output neuron values via a network of weights and hidden neurons. Mathematically, the network is described as follows:

The value (*d_x_*) of neuron *x* is the sum of inputs into *x* from the previous layer of neurons (*Y_i = 1_*_→*n*_ in general; in our example: *I_1_*_→*3*_, *H_1_*_→*2*_). Each of the *n* inputs is a product of value of neuron *Y_i_* and weight of connection between *Y_i_* and *x* (*w_Yi_*_→*x*_).
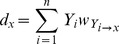
The value of the output (*z_x_*) of a neuron *x* based on its *d_x_* and its threshold *θ_x_* is:
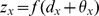
In our case, the function (f) is a sigmoid, where a is a real number constant (optimized for any given network, but generally initially chosen to be between 0.5 and 2).
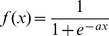
Thus, to compute the output of every neuron in the network we need to use the formula:
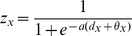
Note, that to compute the output of the *o_neuron* (*z_O_*; the prediction made by the network) we first have to compute the outputs of all *h_neurons* (*z_Hi = 1_*_→*n*_).

In a supervised learning paradigm, experimentally established pairs of inputs and outputs are given to the network during training (Figure 5C). After each input, the network output (*z_O_*) is compared to the observed result (*R*). If the network makes a classification error its weights are adjusted to reflect that error. Establishing the best way to update weights and thresholds in response to error is of the major challenges of neural networks. Many techniques use some form of the delta rule – a gradient descent-based optimization algorithm that makes changes to function variables proportionate to the negative of the approximate gradient of the function at the given point. [It's OK if you didn't understand that sentence – the basic idea is to change the weights and thresholds in the direction opposite of the direction of the error]. In our example, we use the delta rule with back-propagation. This means that to compute the error of the hidden layer, the threshold of the output layer (*θ_O_*) and the weights connecting the hidden layer to the output layer (*w_h1_*_→*O*_, *w_h2_*_→*O*_) need to be changed first.

The steps are as follows:

1. Compute the error (*e_O_*) of *z_O_* as compared to result *R*. Note, that the difference between the expected and the observed values defines the gradient (*g*) at the output neuron.
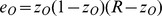

2. Compute the change in the threshold of the output layer (Δ*θ_O_*), using a variable λ, the learning rate constant - a real number, often initialized to 0.1–0.2 and optimized for each network)
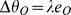

3. Compute the change in the weights connecting the hidden layer to the output, *w_Hi_*_→*O*_.
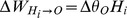

4. Compute the gradient (*g_i_*) at hidden neurons
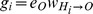
Note, from here all steps are the same as above
5. Compute the error at *z_Hi_*

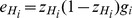

6. Compute the change in *θ_Hi_*

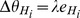

7. Compute the change in *w_Ij_*_→*Hi*_

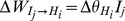

In on-line updating mode of our example, weights and thresholds are altered after each set of input transmissions. Once the network has “seen" the full set of input/output pairs (one epoch/iteration), training continues re-using the same set until the performance is satisfactory. Note that neural networks are sensitive to dataset imbalance. *I.e.* it is preferable to “balance" the training data, such that the number of instances of each class is presented a roughly equal number of times.

In testing, updating of the weights no longer takes place; *i.e.* the *z_O_* for any given set of inputs is constant over time. See Exercise 8 for an experience with testing. Note, there are many variations on the type and parameters of network learning (propagation mode and direction, weight update rules, thresholds for stopping, etc.) Please consult the necessary literature for more information, *e.g.* [134].

## 6. Summary

The development of high throughput technologies has augmented our abilities to identify genetic deficiencies and inconsistencies that lead to the development of diseases. However, a large portion of information in the heaps of data that these methods produce is incomprehensible to the naked eye. Moreover, inferences that could potentially be made from combining different studies and existing research results are beyond reach for anyone of human (not cyborg) descent. Gene prioritization methods (Table 1) have been developed to make sense of this data by extracting and combining the various pieces necessary to link genes to diseases. These methods rely on experimental work such as disease gene linkage analysis and genome wide studies to establish the search space of candidate genes that may possibly be involved in generating the observed phenotype. Further, they utilize mathematical and computational models of disease to filter the original set of genes based on gene and protein sequence, structure, function, interaction, expression, and tissue and cellular localization information. Data repositories that contain the necessary information are diverse in both content and format and require deep knowledge of the stored information to be properly interpreted. Moreover, the models utilizing the various sources assign different weights to the information they extract based on perceived quality and importance of each piece of data available in the context of the entire set of descriptors – a function unlikely to be reproduced in manual data interpretation. Thus, computational gene prioritization techniques serve as interpreters of both of newly retrieved data and of information contained in previous studies. They also are the bridge that connects seemingly unrelated inferences creating an easily comprehensible outlook on an important problem of disease gene annotation.

**Table 1:** The available data sources and gene prioritization tools.

Data Type	Data Content	Possible Sources	Tools
*Experiment, observation*	Linkage, association, pedigree, relevant texts and other data	User provided	CAESAR [140], CANDID [141], ENDEAVOR [122], G2D [15], [16], [17], Gentrepid [142], GeneDistiller [121], PGMapper [143], PRINCE [144], Prioritizer [145], SUSPECTS [146], ToppGene [126], [127]
*Sequence, structure, meta-data*	Sequence conservation, exon number, coding region length, known structural domains and sequence motifs, chromosomal location, protein localization, and other gene-centered information and predictions	SCOP [147], PFam [148], [149], ProSite [150], UniProt, Entrez Gene [151], ENSEMBL [152], InterPro [153], LocDB [154], GeneCards [155], PredictProtein [156]	CAESAR, CANDID, ENDEAVOR, G2D, Gentrepid, GeneDistiller, GeneProspector [125], MedSim [157], MimMiner [158], PGMapper, PhenoPred [159], Prioritizer, PROSPECTR [54], SNPs3D [106], SUSPECTS, ToppGene
*Pathway, protein-protein interaction, genetic linkage, expression*	Disease-gene associations, pathways and gene-gene/protein-protein interactions/interaction predictions, and gene expression data	KEGG [160], [161], STRING, Reactome [162], [163], DIP [164], BioGRID [165], GEO [166], [167], ArrayExpress [168], ReLiance [169]	CAESAR, CANDID, DiseaseNet [170], ENDEAVOR, G2D, Gentrepid, GeneDistiller, GeneWanderer [20], MaxLink [171], MedSim, PGMapper, PhenoPred, PRINCE, Prioritizer, SNPs3D, SUSPECTS, ToppGene
*Non-human data*	Information about related genes and phenotypes in other species	OrthoDisease [172], OrthoMCL [173], MGD [174], Pathbase [175]	CAESAR, CANDID, ENDEAVOR, GeneDistiller, GeneProspector, GeneWanderer, MedSim, Prioritizer, PROSPECTR, SNPs3D, SUSPECTS, ToppGene
*Ontologies*	Gene, disease, phenotype, and anatomic ontologies	GO, DO [176], MPO [177], [178], HPO [179], eVOC [180]	CAESAR, ENDEAVOR, G2D, GeneDistiller, MedSim, PhenoPred, Prioritizer, SNPs3D, ToppGene
*Mutation associations and effects*	Information about existing mutations, their functional and structural effects and their association with diseases, predictions of functional or structural effects for the mutations in the gene in question	dbSNP, PMD [111], GAD, DMDM, SNAP, PolyDoms, SNPdbe, SNPselector, RAVEN, SNPeffect, PHD-SNP, Mutation@A Glance, PromoLign, SIFT, PolyPhen, PupaSNP finder, FASTSNP	CAESAR, CANDID, GeneProspector, GeneWanderer, PROSPECTR, SNPs3D, SUSPECTS
*Literature*	Mixed information of all types extracted from literature references (*e.g.* disease-gene correlation and non-ontology based gene-function assignment)	PubMed, PubMed Central, HGMD [181], GeneRIF, OMIM	CAESAR, CANDID, DiseaseNet, ENDEAVOR, G2D, Gentrepid, GeneDistiller, GeneProspector, GeneWanderer, MedSim, MimMiner, PGMapper, PolySearch [123], PRINCE, Prioritizer, PROSPECTR, SNPs3D, SUSPECTS, ToppGene

There is a wide range of data sources that can be used to infer the above-described pieces of evidence. The existing tools try to take advantage of many (if not all) of them. This table summarizes the collections and methodologies that make current state of the art in gene prioritization possible. Note, not all resources mentioned here are utilized by all gene prioritization tools nor are all data sources available listed. Moreover, some resources may be classified as more than one data-type. Many of the resources reported here are available electronically through the gene prioritization portal [124].

## 7. Exercises

1. Search the GAD (http://geneticassociationdb.nih.gov/) database for all genes reported to be associated with diabetes. Refine this set to find only the positively associated genes. How many are there? Why was the total data set reduced? Count the number of unique diabetes associated genes or explain why this is not feasible. How many SNPs associate these genes with diabetes? Is it realistically possible to experimentally evaluate individual effects of each SNP in this set?
2. Using STRING (http://string-db.org/), find ***all*** genes (hint: use limit of 50) interacting with insulin (confidence >0.99). *Note, this confidence limit is extremely high – computational techniques would normally deal with lower limits and thus larger data sets*. What is the insulin gene name used by STRING? How many interaction partners does your query return? Switch to STRING evidence view. Pick three genes connected to insulin via text mining, but without “insulin" in their full name, and find one reference for each in PubMed (http://www.ncbi.nlm.nih.gov/pubmed/) suggesting that these genes are involved with diabetes. Report Gene IDs (*e.g.* MC4R), PubMed IDs and publication citations. Use PolySearch (http://wishart.biology.ualberta.ca/polysearch) **gene** to **disease** mapping with your gene IDs to do the same. Does your experience confirm that the functional “molecular interaction" evidence works? Why?
3. In AmiGO (GO term browser, http://www.geneontology.org), find the human insulin record (hint: use the insulin ID obtained above). What is the Swiss-Prot ID for insulin? Go to the term view. How many GO term associations does insulin have? Reduce the view to “molecular function" terms. How many terms are left? Create a tree view of these terms (hint: use the “Perform an action" dropdown). Which of the terms is the most exact in defining the likely molecular function of insulin (lowest term in a tree hierarchy)? Display gene products in “GO:0005158: insulin receptor binding", reduce the set to human proteins, and look at the inferred tree. How many gene products are in this term? Pick a set of three gene products (report IDs) and use them to search PolySearch for diabetes associations. In question 3 we used the “common pathway" evidence to show the relationship of genes to diabetes. What type of predictive evidence is used here?
4. Search the Mammalian Phenotype Ontology for keyword “diabetes" and select increased susceptibility (MPO, http://www.informatics.jax.org/searches/MP_form.shtml). How many genotypes are returned? Display the genotypes and click on the Aire^tm1Mand^/Aire^+^ genotype for further exploration. What is the affected gene? Click on gene title (Gene link in Nomenclature section) to display further information. What is an orthologue? What is the human orthologue of your mouse gene? Look up this gene in OMIM (http://www.ncbi.nlm.nih.gov/omim) for association with diabetes. Copy/paste the *citation* from OMIM, describing the gene relationship to diabetes in humans. Do your results confirm the “cross-species" evidence?
5. Search GeneCards (http://www.genecards.org, utilize advanced search) for genes expressing in the pancreas (hint: pancreatic tissue is often affected in diabetes). How many are there? Explore the GeneCard for CCKBR for diabetes association. Do you find that this gene confirms the “disease compartment" evidence? What database, referenced in GeneCards, contains the CCKBR-diabetes association? Now look at the GeneCard of PLEKHG4. Is there evidence for this gene being associated with diabetes (whether in the GeneCards record or otherwise)? Explain your ideas in detail, paying special attention to the “disease compartment" line of evidence.
6. Search UniProt (http://www.uniprot.org) for all reviewed [reviewed:yes] human [organism:“Homo sapiens [9606]"] protein entries that contain natural variants with reference to diabetes [annotation:(type:natural_variations diabetes)]. Use advanced search with specific limits (*i.e.* sequence annotation, natural_variations, term diabetes). How many proteins fit this description? Locate the entry for insulin (identifier from question 3) and find the total number of known coding variants of this sequence. How many are annotated as associated with any form of diabetes? (hint: read the general annotation section for correspondence of abbreviations to types of diabetes). Run SNAP (http://www.rostlab.org/services/snap/) to predict functional effects of all variants. (hint: use comma separated batch submit). How many are predicted to be functionally non-neutral? Do SNAP predictions of functional effect correlate with annotated disease associations? Does this result confirm the “mutant implication" for nsSNPs?
7. Search PolySearch for all genes associated with diabetes. How many results are returned? Look at the PubMed articles that associate “hemoglobin" with diabetes (follow the link from PolySearch). How many are there? Do you find this number large enough to convince you of hemoglobin-diabetes association and why? From reading article titles/extracted sentences, can you identify a biological reason for connecting hemoglobin to diabetes? If one looks especially convincing, cite that article (hint: its OK to not find one). For the first three articles, can you identify a biological reason for connecting hemoglobin to diabetes? Go back to the list of diabetes related genes and look at TCF7L2 articles. Are the biological reasons for matching TCF7L2 to diabetes clearly defined? Cite the most convincing article. Why do you think TCF7L2 is ranked lower in association than hemoglobin? Is there significant evidence for calcium channel (CACNA1E) involvement in diabetes? Consider the PubMed citations. Do you agree with PolySearch classification of this gene-disease association? Does your experience with PolySearch confirm the “text evidence" function of gene prioritization methods?
8. WEKA exercises (choose one).
  Download and install WEKA (http://www.cs.waikato.ac.nz/∼ml/weka/). Using a text-editor (or Microsoft Excel) create comma delimited values (CSV) files identical to the ones described in Figure 5C–D (*i.e.* copy over the training and testing files and replace spaces with commas). Save the files and open the training file in WEKA's Explorer GUI. Open the training file in WEKA's Explorer GUI. You should have four columns of data (Text, Homology, ID, Disease) corresponding to four attributes of each data instance.
  1. Defined Questions: Run the MultiLayer Perceptron with parameters (momentum = 0.5, learning = 0.2, trained using the training set, Figure 5C, repeated 500 times/epochs). Test with the test set (Figure 5D) and output predictions for each test entry (make a screenshot). Assuming that everything predicted below 0 is 0, and everything above is 1. What is your performance (number of true/false positives/negatives, positive/negative accuracy/coverage, overall accuracy)? Try using the Decision Stump classifier with default parameters (take screenshot of output). If everything below 0.5 is 0, and everything above is 1, what is your performance? Is it better or worse than the neural net?
  2. Open ended: Experiment with different tools available from WEKA's Classify section setting the testing set to your test-file's location. First, run the MultiLayer Perceptron with parameters as described in Figure 5, then try to alter the parameters (momentum term, learning rate, and number of epochs). Try using Linear Regression, Decision Table, or Decision Stump classifiers with default parameters. Is your performance on the test set better or worse? Close the WEKA Explorer, reformat your train/test files in the text editor to replace Disease column values by Booleans (True/False) values, and re-open the training file. Use BayesianNet and RandomForest classifiers to test on the testing file. Does you performance improve? Note, that without further understanding of each of the tools, it is nearly impossible to determine which method is applicable to your data.

Answers to the Exercises can be found in Text S1.

**Further Reading**

- Alterovitz G, Ramoni M, eds. (2010) Knowledge-based bioinformatics: from analysis to interpretation. Padstow, Cornwall: John Wiley and Sons Ltd.
- Bromberg Y, Capriotti E, eds. (2012) SNP-SIG 2011: identification and annotation of SNPs in the context of structure, function and disease. Proceedings from SNP-SIG 2011 conference, Vienna, Austria. BMC Genomics 13 Supp 4.
- Chen JY, Youn E, Mooney SD (2009) Connecting protein interaction data, mutations, and disease using bioinformatics. Methods Mol Biol 541: 449–461.
- Dalkilic MM, Costello JC, Clark WT, Radivojac P (2008) From protein-disease associations to disease informatics. Front Biosci 13: 3391–3407.
- Evans JA, Rzhetsky A (2011) Advancing science through mining libraries, ontologies, and communities. J Biol Chem 286: 23659–23666.
- Kann MG (2007) Protein interactions and disease: computational approaches to uncover the etiology of diseases. Brief Bioinform 8: 333–346.
- Krallinger M, Leitner F, Valencia A (2010) Analysis of biological processes and diseases using text mining approaches. Methods Mol Biol 593: 341–382.
- Liberles DA, Teichmann SA, Bahar I, Bastolla U, Bloom J, et al. (2012) The interface of protein structure, protein biophysics, and molecular evolution. Protein Sci 21: 769–785.
- Maulik U, Bandyopadhyay S, Wang JTL, eds. (2010) Computational intelligence and pattern analysis in biological informatics. Hoboken, NJ: John Wiley and Sons, Inc.
- Mooney SD, Krishnan VG, Evani US (2010) Bioinformatic tools for identifying disease gene and SNP candidates. Methods Mol Biol 628: 307–319.
- Moreau Y, Tranchevent LC (2012) Computational tools for prioritizing candidate genes: boosting disease gene discovery. Nat Rev Genet 13: 523–536.
- Oti M, Brunner HG (2007) The modular nature of genetic diseases. Clin Genet 71: 1–11.
- Piro RM, Di Cunto F (2007) Computational approaches to disease-gene prediction: rationale, classification and successes. FEBS J 279: 678–696.

**Glossary**

- **Annotation** – any additional information about a genetic sequence. Annotation types are extremely varied, including functional, structural, regulatory, location-related, organism-specific, experimentally derived, predicted, etc.
- **CNV**, copy number variation – an alteration of the genome, which results in an individual having a non-standard number of copies of one or more DNA sections.
- **Gene prioritization** – the process of arranging possible disease causing genes in order of their likelihood in disease involvement.
- **GWAS**, genome wide association studies – the examination of all genes in the genome to correlate their variation to phenotypic trait variation across individuals in a given population.
- **Genetic linkage** – tendency of certain genetic regions on the same chromosome to be inherited together more often than expected due to limited recombination between them.
- **Genetic marker** – a DNA sequence variant with a known location that can be used to identify specific subsets of individuals (cells, species, individual organisms, etc.).
- **Homologue** – a gene derived from a common ancestor with the reference gene. Generally, gene A is a homologue of gene B if both are derived from a common ancestor.
- **Linkage disequilibrium** – tendency of certain genetic regions (not necessarily on the same chromosome) to be inherited together more often that expected from considering their population frequencies. In reference to gene prioritization, this phenomenon may complicate establishment of causal genes due to their consistent inheritance in complex with non-causal genetic regions.
- **Orthologues** – homologous genes separated by a speciation event. Generally, gene A is an orthologue of gene B if A and B are homologous, but reside in different species. Orthologues often perform the same general function in different organisms.
- **Paralogues** – homologous genes separated by a duplication event (often followed by copy differentiation). Generally, gene A is a paralogue of gene B if A and B are homologous and reside in the same species. A and B can be functionally identical or, on contraire, very different, but are often only slightly dissimilar.
- **Pleiotropy** – the influence of a single gene on a number of phenotypic traits.

## Supporting Information

Text S1 Answers to Exercises.
(DOCX)
