# Supplementary material for: Chapter 15: Disease Gene Prioritization
Source: PLoS Comput Biol. 2013 Apr 25;9(4):e1002902. doi: 10.1371/journal.pcbi.1002902 (PMC3635969; doi:10.1371/journal.pcbi.1002902)
Supplement: Text S1 — Answers to Exercises. (DOCX) [file pcbi.1002902.s001.docx]

# Answers to the Exercises

**1.** Search the GAD (<http://geneticassociationdb.nih.gov/>) database for all genes reported to be associated with diabetes. Refine this set to find only the positively associated genes. How many are there? Why was the total data set reduced? Count the number of unique diabetes associated genes or explain why this is not feasible. How many SNPs associate these genes with diabetes? Is it realistically possible to experimentally evaluate individual effects of each SNP in this set?

***Answer: There are 9370 gene-diabetes associations (one per reference). Of these, 1065 are positively associated. The numbers of SNPs and genes referenced are impossible to compute by hand. This exercise is meant to show that the quantity of experimental data available is too large to deal with manually.***

**2.** Using STRING (http://string-db.org/), find ***all*** genes (hint: use limit of 50) interacting with insulin (confidence > 0.99). *Note, this confidence limit is extremely high – computational techniques would normally deal with lower limits and thus larger data sets.* What is the insulin gene name used by STRING? How many interaction partners does your query return? Switch to STRING evidence view. Pick three genes connected to insulin via text mining, but without “insulin” in their full name, and find one reference for each in PubMed (http://www.ncbi.nlm.nih.gov/pubmed/) suggesting that these genes are involved with diabetes. Report Gene IDs (e.g. MC4R), PubMed ids and publication citations. Use PolySearch (http://wishart.biology.ualberta.ca/polysearch) **gene** to **disease** mapping with your gene IDs to do the same. Does your experience confirm that the functional “molecular interaction” evidence works? Why?

***Answer: April 23, 2012. The insulin gene name is INS and 39 interaction partners are returned. Many, if not all, of these genes (interaction of partners INS) are diabetes associated with evidence in both PubMed and PolySearch. The common pathway evidence suggests that members of the same pathway are often involved in the same disease. Insulin is diabetes related; therefore its interaction partners are also likely diabetes related.***

**3.** In AmiGO (GO term browser, http://www.geneontology.org), find the human insulin record (hint: use the insulin ID obtained above). What is the Swiss-Prot ID for insulin? Go to the term view. How many GO term associations does insulin have? Reduce the view to “molecular function” terms. How many terms are left? Create a tree view of these terms (hint: use the “Perform an action” dropdown). Which of the terms is the most exact in defining the likely molecular function of insulin (lowest term in a tree hierarchy)? Display gene products in “GO:0005158: insulin receptor binding”, reduce the set to human proteins, and look at the inferred tree. How many gene products are in this term? Pick a set of three gene products (report IDs) and use them to search PolySearch for diabetes associations. In question 3 we used the “common pathway” evidence to show the relationship of genes to diabetes. What type of predictive evidence is used here?

***Answer: April 23, 2012. INS_HUMAN is the SP identifier. 67 term associations (4 molecular function terms, either insulin receptor binding or insulin-like growth factor receptor binding should be reported as the lowest). 44 human gene-products should be left for GO:0005158. This is proof of concept for “similar function” evidence – the selected gene products have a function that is similar to that of insulin, which is involved in diabetes.***

**4.** Search the Mammalian Phenotype Ontology for keyword “diabetes” and select increased susceptibility (MPO, <http://www.informatics.jax.org/searches/MP_form.shtml>). How many genotypes are returned? Display the genotypes and click on the Aire^tm1Mand^/Aire^+^ genotype for further exploration. What is the affected gene? Click on gene title (Gene link in Nomenclature section) to display further information. What is an orthologue? What is the human orthologue of your mouse gene? Look up this gene in OMIM (<http://www.ncbi.nlm.nih.gov/omim>) for association with diabetes. Copy/paste the *citation* from OMIM, describing the gene relationship to diabetes in humans. Do your results confirm the “cross-species” evidence?

***Answer: April 23, 2012. 84 genotypes are returned. The affected gene is Aire (tm1mand), the human orthologue is AIRE (orthologue is a gene separated by a speciation event). Citation:* Nithiyananthan, R. et al. [**[**139**](#_ENREF_139)**]. *The diabetes associations for AIRE in human are dubious. This specific citation says that the association doesn’t exist.***

**5.** Search GeneCards (<http://www.genecards.org>, utilize advanced search) for genes expressing in the pancreas (hint: pancreatic tissue is often affected in diabetes). How many are there? Explore the GeneCard for CCKBR for diabetes association. Do you find that this gene confirms the “disease compartment” association? What database, referenced in GeneCards, contains the CCKBR-diabetes association? Now look at the GeneCard of PLEKHG4. Is there evidence for this gene being associated with diabetes (whether in the GeneCards record or otherwise)? Explain your ideas in detail, paying special attention to the “disease compartment” association line of evidence.

***Answer: April 23, 2012. 1511 genes expressed in the pancreas. CCKBR is diabetes associated (PharmGKB), PLEKHG4 is not. It should be clear that not all genes expressed in the pancreas have to be diabetes associated. Extra credit for statements that point out that 1) some diabetes related genes are not expressed in the pancreas, and 2) gene prioritization tools should take all sources of evidence into account.***

**6.** Search UniProt (<http://www.uniprot.org>) for all reviewed [reviewed:yes] human [organism:"Homo sapiens [9606]"] protein entries that contain natural variants with reference to diabetes [annotation:(type:natural_variations diabetes)]. Use advanced search with specific limits (i.e. sequence annotation, natural_variations, term diabetes). How many proteins fit this description? Locate the entry for insulin (identifier from question 3) and find the total number of known coding variants of this sequence. How many are annotated as associated with any form of diabetes? (hint: read the general annotation section for correspondence of abbreviations to types of diabetes). Run SNAP (<http://www.rostlab.org/services/snap/>) to predict functional effects of all variants. (hint: use comma separated batch submit). How many are predicted to be functionally non-neutral? Do SNAP predictions of functional effect correlate with annotated disease associations? Does this result confirm the “mutant implication” for nsSNPs?

***Answer:*** ***April 23, 2012. There are 23 proteins in this category. INS_HUMAN contains 27 coding variants. 22 (or 24, depending on annotation counts) of these are in one way or another diabetes associated. The one non-disease mutant (L68M) is correctly predicted neutral by SNAP. Of the remaining mutants (all disease associated), 25 are predicted to be non-neutral with respect to function by SNAP. Two diabetes-associated mutation are misdiagnosed by SNAP as neutral. These results confirm mutation implication, but one has to be careful when interpreting functional effect as disease.***

**7.** Search PolySearch for all genes associated with diabetes. How many results are returned? Look at the PubMed articles that associate “hemoglobin” with diabetes (follow the link from PolySearch). How many are there? Do you find this number large enough to convince you of hemoglobin-diabetes association and why? From reading article titles/extracted sentences, can you identify a biological reason for connecting hemoglobin to diabetes? If one looks especially convincing, cite that article (hint: its OK to not find one). For the first three articles, can you identify a biological reason for connecting hemoglobin to diabetes? Go back to the list of diabetes related genes and look at TCF7L2 articles. Are the biological reasons for matching TCF7L2 to diabetes clearly defined? Cite the most convincing article. Why do you think TCF7L2 is ranked lower in association than hemoglobin? Is there significant evidence for calcium channel (CACNA1E) involvement in diabetes? Consider the PubMed citations. Do you agree with PolySearch classification of this gene-disease association? Does your experience with PolySearch confirm the “text implication” function of gene prioritization methods?

***Answer: April 23, 2012. 182 results are returned. There are 95 hemoglobin-diabetes articles are numerous, but not very substantial in evidence for association. TCF7L2 articles are few (6), but very much biologically relevant (e.g. the paper entitled: Functional analysis of TCF7L2 genetic variants associated with type 2 diabetes.) Text mining algorithms are blind to biological evidence, but value direct associations. Thus, TCF7L2 comes in lower than hemoglobin because there are fewer articles. However, it is higher than other possible culprits, because the text-based evidence is clearer. The calcium channel paper directly relates the gene to the disease. Text-based associations work best for high numbers articles, but are also ok for a small number of highly relevant citations.***

**8. WEKA exercises (choose one).**

Download and install WEKA (<http://www.cs.waikato.ac.nz/~ml/weka/>). Using a text-editor (or Microsoft Excel) create comma delimited values (CSV) files identical to the ones described in Figure 5C-D (i.e. copy over the training and testing files and replace spaces with commas). Save the files and open the training file in WEKA’s Explorer GUI. Open the training file in WEKA’s Explorer GUI. You should have four columns of data (Text, Homology, ID, Disease) corresponding to four attributes of each data instance.

**8.1 Defined Questions:** Run the MultiLayer Perceptron with parameters (momentum=0.5, learning=0.2, trained using the training set, Figure 5C, repeated 500 times/epochs). Test with the test set (Figure 5D) and output predictions for each test entry (make a screenshot). Assuming that everything predicted below 0 is 0, and everything above is 1. What is your performance (number of true/false positives/negatives, positive/negative accuracy/coverage, overall accuracy)? Try using the Decision Stump classifier with default parameters (take screenshot of output). If everything below 0.5 is 0, and everything above is 1, what is your performance? Is it better or worse than the neural net?

***Answers:***

***Neural net: TP = 1, FP = 0, TN = 3, FN =3. PA=1, PC=0.25,NA=0.5,NC=1, Q2=4/7(0.57)***

***inst# actual predicted error***

***1 1 -0.073 -1.073***

***2 1 -0.016 -1.016***

***3 1 0.951 -0.049***

***4 0 -0.091 -0.091***

***5 1 -0.148 -1.148***

***6 0 -0.015 -0.015***

***7 0 -0.067 -0.067***

***Decision stump: TP=2, FP=0, TN=3, FN=2. PA=1, PC=0.5, NA=3/5(0.6), NC=1, Q2=5/7(0.72)***

***inst# actual predicted error***

***1 1 0 -1***

***2 1 0 -1***

***3 1 0.8 -0.2***

***4 0 0 0***

***5 1 0.8 -0.2***

***6 0 0 0***

***7 0 0 0***

**8.2** Open ended: Experiment with different tools available from WEKA’s Classify section setting the testing set to your test-file’s location. First, run the MultiLayer Perceptron with parameters as described in Figure 5, then try to alter the parameters (momentum term, learning rate, and number of epochs). Try using Linear Regression, Decision Table, or Decision Stump classifiers with default parameters. Is your performance on the test set better or worse? Close the WEKA Explorer, reformat your train/test files in the text editor to replace Disease column values by Booleans (True/False) values, and re-open the training file. Use BayesianNet and RandomForest classifiers to test on the testing file. Does you performance improve? Note, that without further understanding of each of the tools, it is nearly impossible to determine which method is applicable to your data.
